# NG2 proteoglycan-dependent recruitment of tumor macrophages promotes pericyte-endothelial cell interactions required for brain tumor vascularization 

**DOI:** 10.1080/2162402X.2014.1001204

**Published:** 2015-01-22

**Authors:** Fusanori Yotsumoto, Weon-Kyoo You, Pilar Cejudo-Martin, Karolina Kucharova, Kenji Sakimura, William B Stallcup

**Affiliations:** 1 Sanford-Burnham Medical Research Institute; Cancer Center; La Jolla, CA USA; 2 Department of Biochemistry; Faculty of Medicine; Fukuoka University, Fukuoka, Japan; 3 Biologics Business; Research and Development Center; Hanwha Chemical; Daejeon, South Korea; 4 Department of Cellular Neurobiology; Brain Research Institute; Niigata University, Niigata, Japan

**Keywords:** macrophage recruitment, NG2 proteoglycan, pericyte-endothelial cell interaction, tumor vascularization, tumor microenvironment

## Abstract

Early stage growth of intracranial B16F10 tumors is reduced by 87% in myeloid-specific NG2 null (Mac-NG2ko) mice and by 77% in pericyte-specific NG2 null (PC-NG2ko) mice, demonstrating the importance of the NG2 proteoglycan in each of these stromal compartments. In both genotypes, loss of pericyte-endothelial cell interaction results in numerous structural defects in tumor blood vessels, including decreased formation of endothelial cell junctions and decreased assembly of the vascular basal lamina. All vascular deficits are larger in Mac-NG2ko mice than in PC-NG2ko mice, correlating with the greater decrease in pericyte-endothelial cell interaction in Mac-NG2ko animals. Accordingly, tumor vessels in Mac-NG2ko mice have a smaller diameter, lower degree of patency, and higher degree of leakiness than tumor vessels in PC-NG2ko mice, leading to less efficient tumor blood flow and to increased intratumoral hypoxia. While reduced pericyte interaction with endothelial cells in PC-NG2ko mice is caused by loss of NG2-dependent pericyte activation of β1 integrin signaling in endothelial cells, reduced pericyte-endothelial cell interaction in Mac-NG2ko mice is due to a 90% reduction in NG2-dependent macrophage recruitment to tumors. The absence of a macrophage-derived signal(s) in Mac-NG2ko mice results in the loss of pericyte ability to associate with endothelial cells, possibly due to reduced expression of N-cadherin by both pericytes and endothelial cells.

## Introduction

Pro-tumorigenic macrophages are extremely versatile and powerful components of the tumor microenvironment. In addition to suppressing antitumor immunity, tumor macrophages also promote tumor cell growth, migration, invasion, vascular intravasation and extravasation, and survival in metastatic sites.^1,2^ In this latter context, macrophages are also thought to be responsible for conditioning of pre-metastatic sites. In addition, macrophages have important roles in promoting tumor vascularization.^3,4^ A growing body of evidence suggests that different macrophage subpopulations are responsible for these varied functions, although these subpopulations are for the most part not yet well defined. We have previously suggested that the NG2 proteoglycan plays a role in defining tumor macrophage specification. In the context of the mouse mammary tumor virus-polyoma middle T (MMTV-PyMT) model of breast cancer,^5^ germline ablation of NG2 retards mammary tumor progression.^6^ While we attributed an important part of this effect to loss of NG2 from microvascular pericytes, with resulting deficits in mammary tumor vascularization, we also noted substantial losses of both tumor-associated macrophages (TAMs) and Tie2-expressing monocytes (TEMs) in tumors in NG2 null mice. In light of the importance of these myeloid populations in tumor vascularization,^7-9^ one of our goals has been to investigate the role of NG2 in macrophage-dependent tumor vascularization.

Several reports from our laboratory have focused on pericyte function during tumor vascularization,^6,10-12^ especially with regard to their interaction with endothelial cells. These studies were aided substantially by development of methodologies for quantifying key aspects of tumor vessel structure and function. Coupled with our adoption of Cre-lox methods for cell type-specific ablation of NG2 in mice,^12,13^ these techniques have allowed us to demonstrate that pericyte-specific NG2 ablation impairs vascularization of intracranial B16F10 melanoma tumors via loss of NG2-mediated activation of β1 integrin signaling in closely-apposed vascular endothelial cells. Loss of this key aspect of pericyte-endothelial cell crosstalk leads to defects in other aspects of vessel structure, including assembly of endothelial junctions, deposition of the vascular basement membrane, and formation of patent vessels.^12^

We now extend this approach to examination of the effects of myeloid-specific NG2 ablation on intracranial B16F10 tumor vascularization and growth. Myeloid-specific ablation of NG2 (Mac-NG2ko) retards early brain tumor growth to a greater extent than the pericyte-specific NG2 ablation (PC-NG2ko). This is correlated with more extensive vascular defects in Mac-NG2ko mice than in PC-NG2ko mice. Mac-NG2ko tumors are characterized by a 90% reduction in tumor macrophages, accompanied by apparent loss of a macrophage-derived signal that promotes pericyte association with endothelial cells. This reduction in pericyte-endothelial cell interaction is more severe than that observed in PC-NG2ko mice, leading to the more extensive vascular abnormalities observed in Mac-NG2ko mice.

## Results

### Myeloid- and pericyte-specific ablation of NG2 both retard brain tumor progression

**Figure 1.**
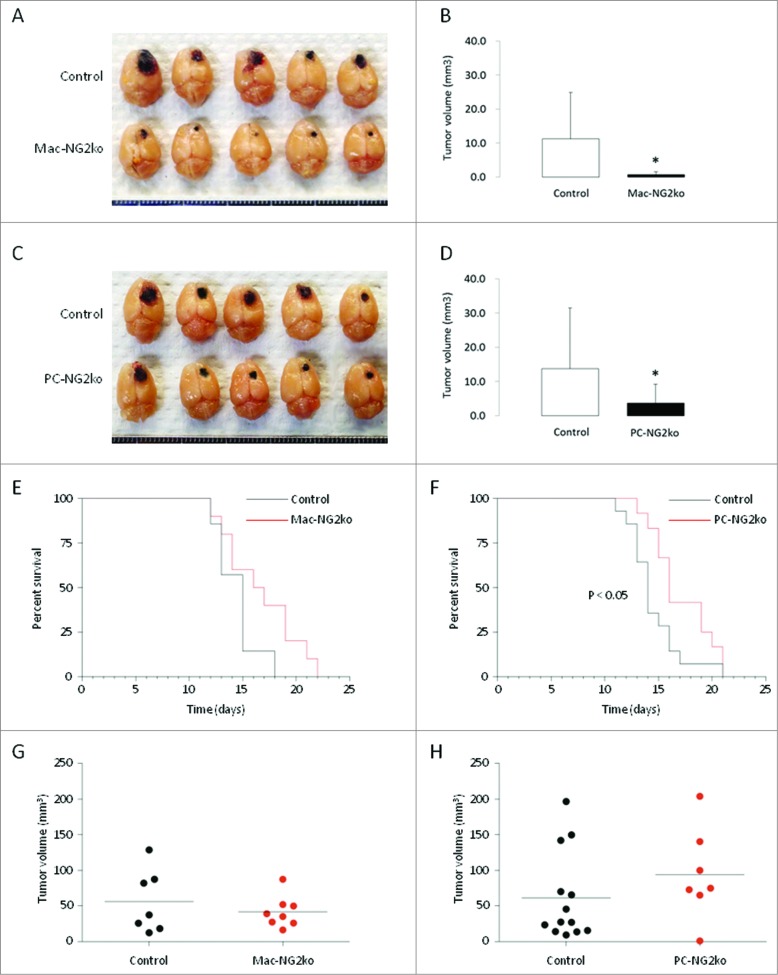
**Decreased brain tumor progression in myeloid- and pericyte-specific NG2 null mice.** B16F10 tumors in control versus Mac-NG2ko mice (**A**) and in control vs. PC-NG2ko mice (**C**) at 10 d after tumor initiation. Graphs quantify tumor volumes in control versus Mac-NG2ko mice (**B**) and in control vs. PC-NG2ko mice (**D**). Data in B represent 21 control and 20 Mac-NG2ko mice. Data in D represent 22 control and 18 PC-NG2ko mice. *p < 0.01 compared to controls. (**E, F**). Kaplan–Meier survival curves for control (*n* = 7) versus Mac-NG2ko (*n* = 10) and control (*n* = 14) vs. PC-NG2ko (*n* = 12) mice (*p* < 0.5). (**G, H**). Terminal tumor volumes for control versus Mac-NG2ko mice and for control vs. PC-NG2ko mice. Each data point represents one mouse. Horizontal lines indicate mean tumor volumes.

To define the respective roles of NG2 in pericyte and macrophage contributions to brain tumor growth, we used Cre/Lox technology to generate pericyte- and myeloid-specific NG2 null mice. NG2 floxed mice were crossed with Pdgfrb-Cre transgenic mice for pericyte-specific ablation of NG2 (PC-NG2ko), and with LysM-Cre transgenic mice for Mac-NG2ko. As described previously,^10-12^ NG2-negative B16F10 melanoma cells were microinjected intracranially into the subcortical white matter (corpus callosum) of control mice and both lines of NG2 null mice, and the sizes of brain tumors were measured at 10 d after injection. Significant reductions of early tumor growth are observed in both PC-NG2ko and Mac-NG2ko mice (**Figs. 1A and C**). The combined data from three trials with each genotype reveal an 87% decrease in tumor volume in Mac-NG2ko mice versus a 77% decrease in tumor volume in PC-NG2ko mice (**Figs. 1B and D**). **Fig. S1A** shows that differences in tumor volume are statistically significant in each of the three individual trials with Mac-NG2ko mice. For PC-NG2ko mice, only individual trial 3 yields statistically significant differences in tumor volume (**Fig. S1B**). However, the combination of all three trials in PC-NG2ko mice provides statistically significant differences in tumor volume (**Fig. 1D**). These results suggest that both macrophage-specific and pericyte-specific NG2 ablation significantly retard tumor progression, but that the impact of macrophage-specific NG2 ablation may be larger than that of pericyte-specific NG2 ablation.

Tests of life span in tumor-bearing mice reveal that Mac-NG2ko mice exhibit a small trend toward increased survival compared to control mice (**Fig. 1E**). However, this difference is not statistically significant. A second trial yielded similar results. This is surprising in light of the large impact on tumor size in these mice at the 10-d time point. In contrast, PC-NG2ko mice have a small, but statistically significant survival advantage over control mice. The time for median survival is extended by 2 d (about 15%) in PC-NG2ko mice (**Fig. 1F**). Terminal tumor volumes are very similar in all three genotypes (**Figs. 1G, H**).

### Myeloid-specific NG2 ablation impairs macrophage recruitment to tumors

**Figure 2.**
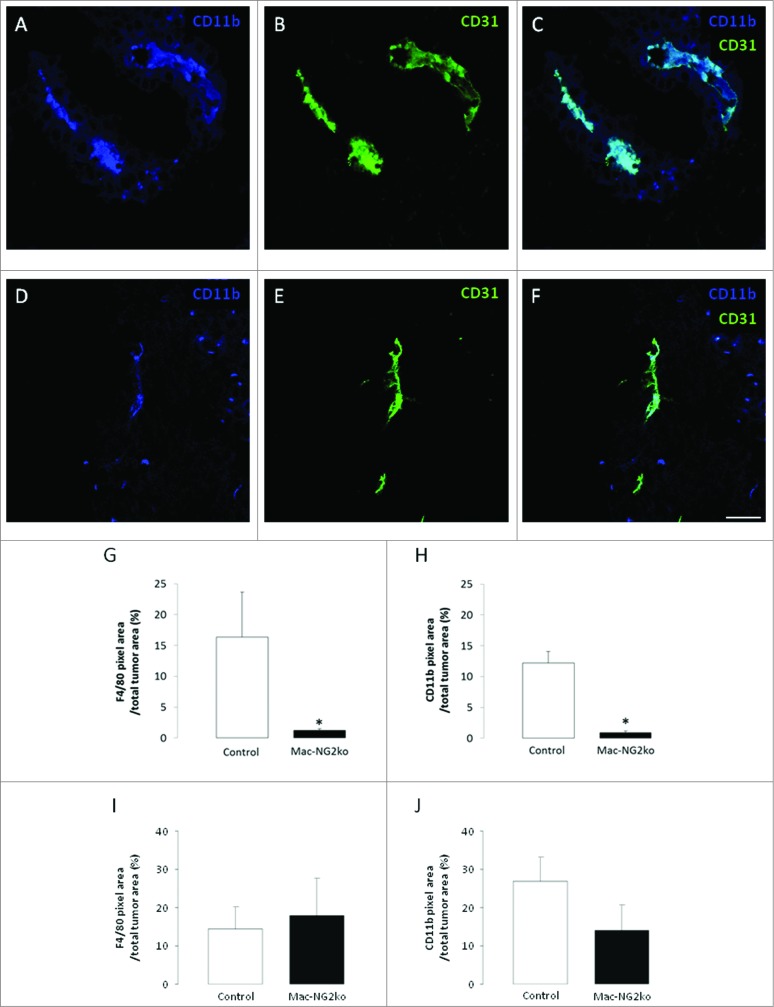
**Myeloid-specific NG2 ablation impairs macrophage recruitment to tumors.** Double immunostaining for CD11b (blue) and CD31 (green) in tumor sections from control (**A–C**) and Mac-NG2ko mice (**D–F**). Areas of overlap in z-stacks appear as pale blue (**C,F**). Quantification of macrophage abundance in tumors using the markers F4/80 (**G, I**) and CD11b (**H, J**). Data are plotted as the percentage of total tumor area occupied by marker pixels in 10-d tumors (**G, H**) and 16-day tumors (**I, J**). Scale bars = 20 μm. *p < 0.01 compared to controls.

To examine macrophage abundance in B16F10 tumors in control, Mac-NG2ko, and PC-NG2ko mice, we used immunolabeling for the macrophage markers F4/80 (EGF-TM7 family), CD11b (αM integrin subunit), and CD18 (β2 integrin subunit). In tumors in control mice, immunolabeling for CD11b (**Figs. 2A–C**), F4/80 (**Figs. S2A–C**), and CD18 (**Figs. S2G–I**) all reveal very large perivascular populations of TAMs . In tumors in Mac-NG2ko mice, the same three macrophage markers identify populations of TAMs (**Figs. 2D–F**; **Figs. S2D–F; J–L**) that are reduced more than 10-fold compared to tumors in control mice (**Figs. 2G, H**). This is not due to a decrease in the number of circulating macrophages in Mac-NG2ko mice. Flow cytometric analysis shows that CD11b-positive macrophages comprise 16% of circulating cells in control mice vs. 18% of circulating cells in Mac-NG2ko mice. The fact that macrophage recruitment is not impaired in PC-NG2ko mice (**Figs. S3A–C**) emphasizes the unique effect of myeloid-specific NG2 ablation on macrophage recruitment. In contrast to these data from 10-d tumors, tumors examined at 16 d in Mac-NG2ko mice fail to exhibit a statistically significant decrease in the abundance of F4/80-positive (**Fig. 2I**) or CD11b-positive cells (**Fig. 2J**). This suggests that macrophage recruitment is impaired, but not completely abolished by NG2 ablation, so that the abundance of tumor macrophages substantially recovers at later time points in Mac-NG2ko mice.

We previously demonstrated the pericyte-specific ablation of NG2 in PC-NG2ko mice.^12^ To confirm the specificity of NG2 ablation in macrophages in Mac-NG2ko mice, we performed triple immunostaining for NG2, PDGFRβ, and CD11b (**Figs. S4A–F**). In tumors in control mice, NG2 expression is seen in both PDGFRβ-positive pericytes (**Figs. S4G, H**, arrowheads) and CD11b-positive macrophages (**Figs. S4K, L**, arrows). In tumors in Mac-NG2ko mice, NG2 is still present on PDGFRβ-positive pericytes (**Figs. S4I, J**, arrowheads), but is missing from macrophages (**Figs. S4M, N**). Macrophage expression of NG2 is quite transient compared to NG2 expression by other cell types. This makes it difficult not only to provide quantitative values for the efficiency of NG2 ablation in macrophages, but also to quantify the sizes of various macrophage subpopulations that express NG2. Nevertheless, our data indicate that NG2 expression is ablated from the large majority of CD11b-positive macrophages in Mac-NG2ko mice.

### NG2-dependent recruitment of tumor macrophages derived from bone marrow

**Figure 3.**
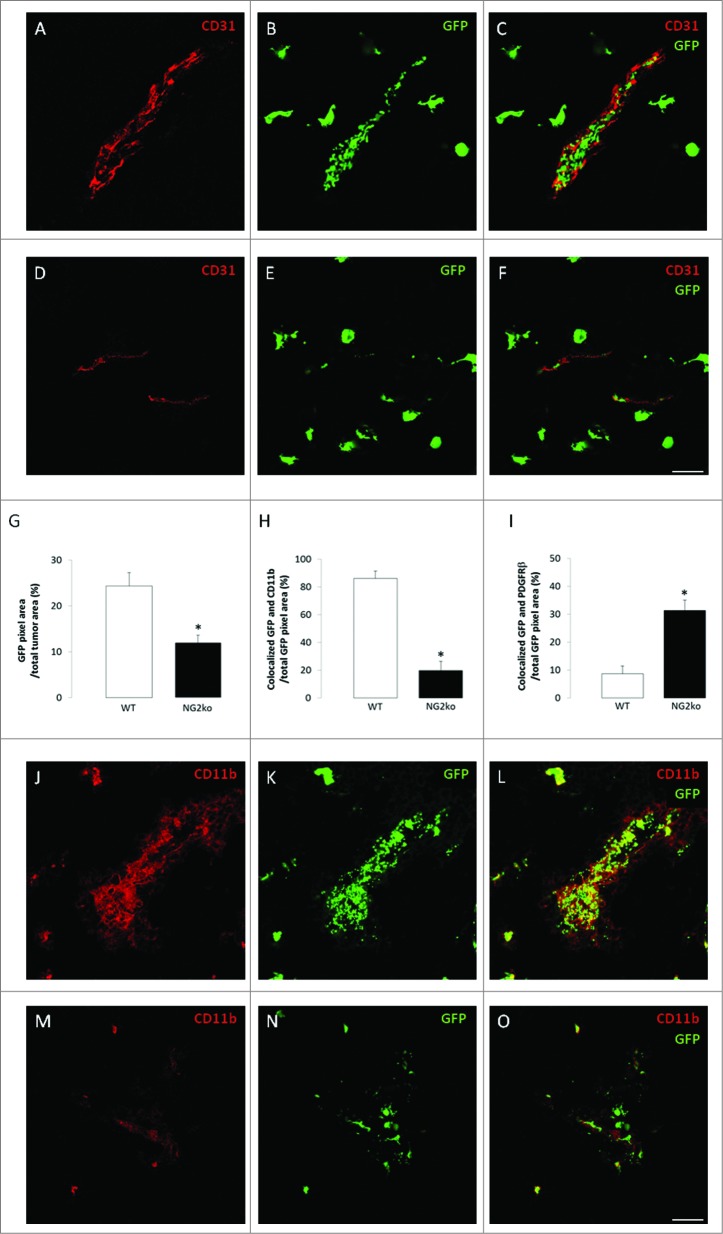
**NG2-dependent recruitment of bone marrow-derived macrophages.** Tumor sections from mice transplanted with EGFP-positive bone marrow from wild type (**A–C**) or germline NG2 null donors (**D–F**) were used to localize bone marrow-derived macrophages in relation to CD31-positive blood vessels (red). Panels C and F are merged images of confocal z-stacks. (**G**) Quantification of EGFP pixels as a percentage of tumor area. In parallel, sections were immunostained for CD11b (red) and for PDGFRβ (not shown) to identify EGFP-positive macrophages and pericytes, respectively, in mice transplanted with EGFP-positive bone marrow from wild type (**J–L**) or germline NG2 null donors (**M–O**). Merged images of confocal z-stacks are shown in panels L and O. (**H**) Quantification of the percentage of EGFP-positive cells that are CD11b-positive. (**I**) Quantification of the percentage of EGFP-positive cells that are PDGFRβ-positive. Scale bars = 20 μm. *p < 0.01 compared to controls.

In order to confirm the results obtained via use of macrophage markers, and also to examine the relative contributions of bone marrow-derived macrophages vs. tissue-resident myeloid cells (microglia), we established B16F10 tumors in the brains of wild type mice that had been irradiated and then transplanted with bone marrow cells from either EGFP-positive NG2^+/+^ or EGFP-positive NG2^−/−^ transgenic donors.^6^ EGFP-positive cells are very abundant in tumors established in mice receiving NG2^+/+^ bone marrow (**Figs. 3A–C**). In contrast, the abundance of EGFP-positive cells is reduced by 50% in tumors established in mice receiving NG2^−/−^ bone marrow (**Figs. 3D–G**). In tumors in mice receiving NG2^+/+^ bone marrow, more than 80% of the EGFP-positive cells are labeled for CD11b (**Figs. 3H, J–L**), establishing their myeloid identity. 10% of EGFP-positive cells in these tumors express PDGFRβ (**Fig. 3I**), showing that tumor pericytes can also be derived from bone marrow. Because NG2 ablation diminishes macrophage recruitment, but not pericyte recruitment, the relative abundance of EGFP-positive macrophages and pericytes is reversed in tumors in mice receiving NG2^−/−^ bone marrow. The relative abundance of EGFP-positive cells expressing CD11b drops to 20% (**Figs. 3H, M–O**), while the relative abundance of EGFP-positive, PDGFRβ positive cells increases to 30% in these tumors (**Fig. 3I**). In addition to confirming that NG2 ablation reduces myeloid cell recruitment to tumors, these bone marrow transplantation results also suggest that tumor myeloid cells are largely macrophage rather than microglia in nature.

Comparison of tumor volumes in mice receiving NG2^+/+^ bone marrow vs. NG2^−/−^ bone marrow reveal a 4-fold decrease in tumor size in the NG2 deficient mice (**Figs. S5 A, B**). The fact that this effect on tumor volume is smaller than that noted in Mac-NG2ko mice is likely due to the incomplete level of bone marrow reconstitution in our transplants. We routinely achieve 75–80% engraftment, leaving a significant endogenous NG2^+/+^ population even after transplantation of NG2^−/–^ bone marrow.

### Macrophage-specific NG2 ablation impairs tumor vascularization

**Figure 4.**
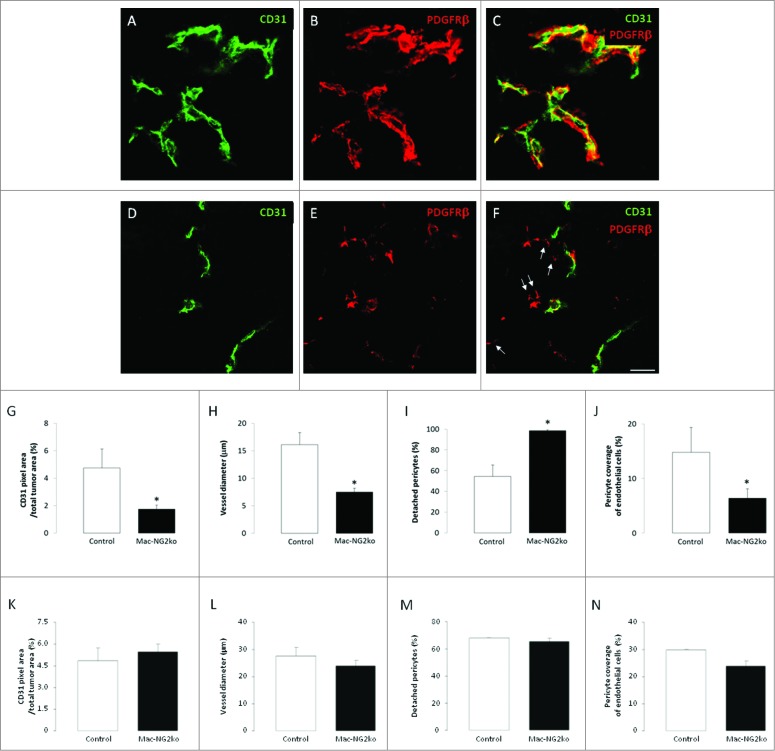
**Loss of pericyte-endothelial cell interaction following myeloid-specific NG2 ablation.** Double immunostaining for PDGFRβ (red) and CD31 (green) was used to quantify pericyte and endothelial cell abundance, along with pericyte ensheathment of endothelial cells, in tumor sections from control (**A–C**) and Mac-NG2ko mice (**D–F**). Z-stacks of confocal images were used to generate three-dimensional reconstructions for this purpose. 10-d tumors in Mac-NG2ko mice exhibit decreased CD31 pixel density (**G**) due to diminished vessel diameter (**H**). In addition, the percentage of pericytes lacking physical contact with endothelial cells (**I**; arrows in **F**) is increased, resulting in diminished pericyte ensheathment of endothelial cells (**J**). In 16-d tumors in Mac-NG2ko mice, CD31 pixel density (**K**), vessel diameter (**L**), detached pericytes (**M**), and pericyte-endothelial cell ensheathment (**N**) all approach levels seen in control mice. Scale bar = 20 μm. *p < 0.01 compared to controls.

We previously showed that PC-NG2ko impairs the ability of pericytes to interact with endothelial cells, resulting in several structural and functional deficits in tumor blood vessels.^12^ In light of the importance of macrophages in tumor vascularization, we examined vessel structure and function in B16F10 tumors grown in control vs. Mac-NG2ko mice. We used double immunolabeling for PDGFRβ and CD31 to compare pericyte-endothelial cell relationships in these tumors (**Figs. 4A–F**). While the density of CD31 labeling per vessel and the number of vessels per tumor area (quantification not shown) are not altered in tumors in Mac-NG2ko mice, the overall density of CD31 labeling is reduced in these tumors due to the pronounced decrease in vessel diameter (**Figs. 4G, H**). Strikingly, although macrophage-specific NG2 ablation does not alter the overall abundance of pericytes in tumors (data not shown), the number of pericytes lacking physical association with CD31-positive endothelial cells increases 2-fold (**Figs. 4F and I** arrows). Thus, even though pericyte abundance is not reduced and pericytes still express NG2, macrophage-specific ablation of NG2 substantially diminishes pericyte coverage of endothelial cells (**Figs. 4C, F, J**).

In 16-d tumors, in correspondence with increased macrophage recruitment, vascular parameters in Mac-NG2ko mice more closely match those of control mice. The density of CD31 labeling is similar in tumors in control and Mac-NG2ko mice (**Fig. 4K**), in agreement with the similar diameter of vessels in the two sets of tumors (**Fig. 4L**). Consistent with these observations, pericyte-endothelial cell relationships are also normalized in 16-d tumors in Mac-NG2ko mice. Pericyte association with and ensheathment of endothelial cells are both similar in Mac-NG2ko and control mice (**Figs. 4M, N**).

**Figure 5.**
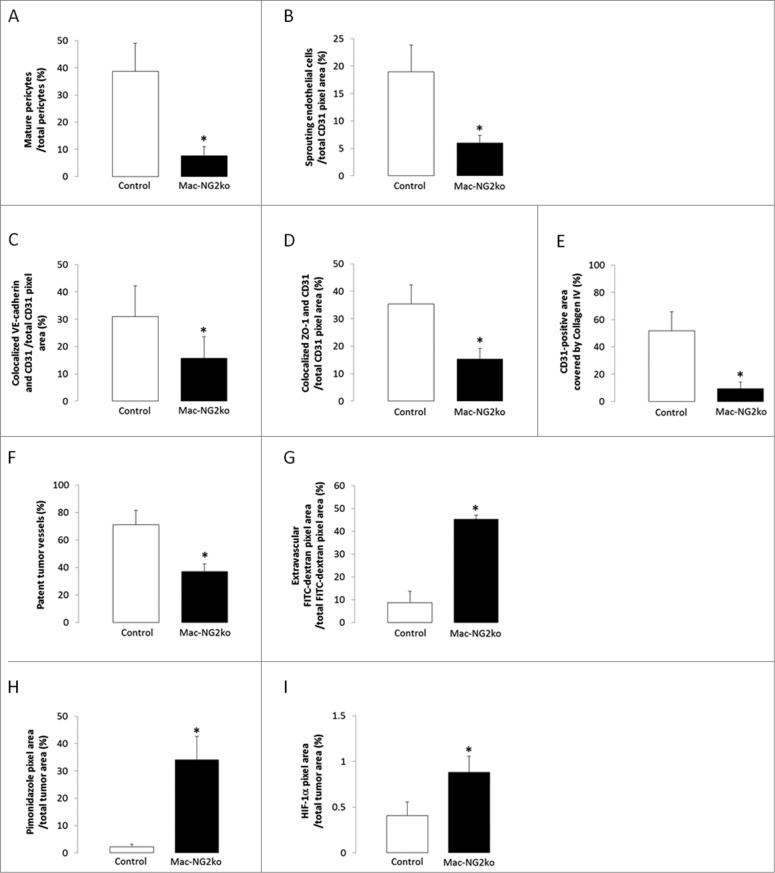
**Structural and functional changes in tumor vessels following myeloid-specific NG2 ablation. *Vessel structure.*** (**A**) Double immunostaining for α-SMA and PDGFRβ was used to determine the percentage of mature pericytes in control versus Mac-NG2ko tumor vessels. (**B**) CD31 and VEGFR3 double immunostaining was used to quantify the abundance of sprouting endothelial cells as a function of total vessel area. (**C and D**) Double immunostaining for CD31 and either VE-cadherin or ZO-1 was used to quantify the abundance of endothelial cell junctions as a function of total vessel area. (**E**) Assembly of the vascular basement membrane was assessed via double immunostaining for CD31 and collagen IV. *p < 0.01 compared to controls. ***Vessel function***. (**F**) Immunostaining for CD31 in FITC-LEA perfused vessels was used to determine the percentage of patent vessels in tumors from control and Mac-NG2ko mice. (**G**) Immunostaining for CD31 was used to quantify the percentage of perfused FITC-dextran located external to tumor vessels in control and Mac-NG2ko mice. (**H**) The extent of intratumoral hypoxia was determined via quantification of bound pimonidazole in tumors from control and Mac-NG2ko mice. (**I**) Levels of HIF-1α were determined by immunostaining with a specific HIF-1α antibody in tumors from control and Mac-NG2ko mice. *p < 0.01 vs. control.

Since we have found that diminished interactions between pericytes and endothelial cells lead to other types of vascular deficits,^10,12^ we further characterized the properties of tumor vessels in Mac-NG2ko mice. Loss of pericyte-endothelial cell communication alters the biology of both of these cell types. Pericyte maturation is reduced 5-fold in tumors in Mac-NG2ko mice, as judged by the percentage of PDGFRβ-positive pericytes expressing the α-smooth muscle actin (SMA) maturation marker (**Fig. 5A**). In parallel, endothelial cell sprouting is reduced 3-fold, as measured by VEGFR3 expression on CD31-labeled endothelial cells (**Fig. 5B**). VEGFR3-positive cells do not express LYVE-1, confirming their identity as sprouting endothelial cells rather than lymphatic endothelial cells (**Figs. S6A–F**). In addition, endothelial junction formation is decreased 2-fold in Mac-NG2ko mice, as judged by double labeling for CD31 and for either of two junctional proteins, VE-cadherin and ZO-1 (**Figs. 5C, D**). Finally, assembly of the vascular basement membrane, an activity requiring the participation of both pericytes and endothelial cells, is reduced 8-fold as a result of diminished pericyte-endothelial cell interaction. This is assessed by quantifying overlap between CD31 labeling and labeling for collagen IV, the major collagen species present in the vascular basal lamina (**Fig. 5E**).

**Figure 6.**
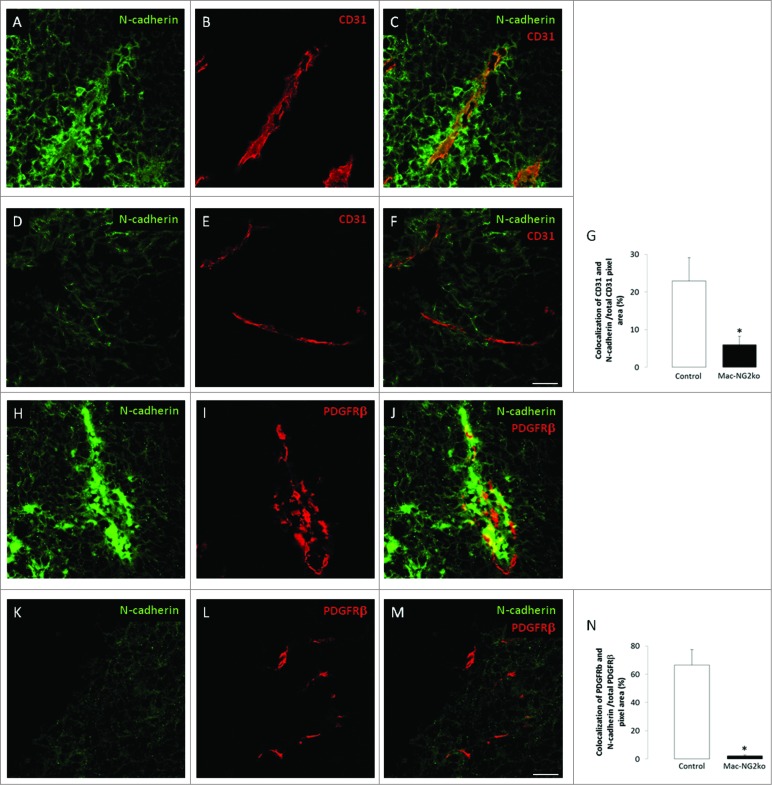
**Loss of vascular N-cadherin expression following myeloid-specific ablation of NG2.** Double immunostaining for CD31 (red) and N-cadherin (green) was used to assess endothelial cell expression of N-cadherin in control (**A–C**) and Mac-NG2ko tumor vessels (**D–F**). (**G**) Quantification of N-cadherin/CD31 colocalization as a percentage of total CD31. Double immunostaining for PDGFGRβ (red) and N-cadherin (green) was used to evaluate pericyte expression of N-cadherin in control (**H–J**) and Mac-NG2ko tumor vessels (**K–M**). (**N**) Quantification of N-cadherin/PDGFRβ colocalization as a percentage of total PDGFRβ. Scale bars = 20 μm. *p < 0.01 compared to controls.

**Figure 7.**
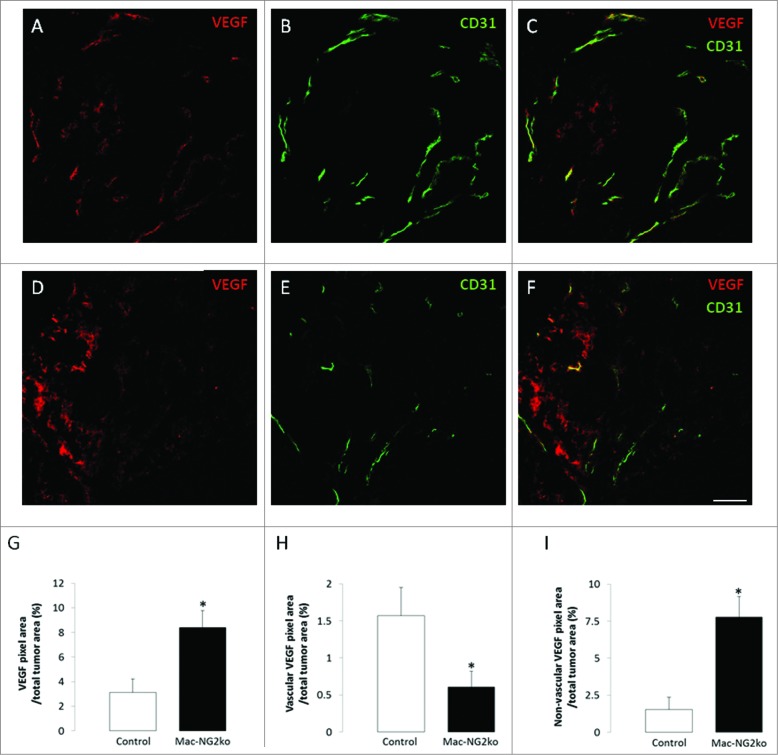
**Altered expression of VEGF-A following myeloid-specific ablation of NG2.** Double immunostaining for VEGF-A (red) and CD31 (green) was used to quantify and localize VEGF-A expression in tumors from control (**A–C**) and Mac-NG2ko tumors (**D–F**). Overall expression of VEGF-A increases 3-fold in Mac-NG2ko tumors (**G**), consistent with the increased HIF-1α expression seen in **Fig. 5I**. However, VEGF-A in control tumors is highly localized to blood vessels, while vascular VEGF-A in Mac-NG2ko tumors is reduced 3-fold (**H**). Instead, non-vascular VEGF-A in Mac-NG2ko tumors is increased by a factor of 5 (**I**). Scale bars = 60 μm. *p < 0.01 compared to controls.

Since expression of VE-cadherin is influenced by N-cadherin,^14^ and since N-cadherin is a key mediator of adhesion between pericytes and endothelial cells,^15,16^ we also examined N-cadherin expression in Mac-NG2ko tumors. N-cadherin levels are greatly reduced in both endothelial cells and pericytes in these tumors (**Figs. 6A–F and H–M**). Endothelial cell expression of N-cadherin is reduced almost 5-fold (**Fig. 6G**), while pericyte N-cadherin expression decreases by a factor of 20 (**Fig. 6N**). In contrast, N-cadherin expression by endothelial cells and pericytes is unaffected by PC-NG2ko (**Figs. 7A–N**), suggesting that loss of N-cadherin is a cause rather than the result of diminished pericyte-endothelial cell interaction.

Based on our previous experience with germline NG2 null mice and PC-NG2ko mice,^6,10,12^ we expected these structural deficits in tumor vessels to have negative effects on vessel function in Mac-NG2ko mice. We therefore quantified vessel patency, vessel leakiness, and hypoxia in tumors from control and Mac-NG2ko mice. The percentage of patent vessels falls from 70% in tumors in control mice to 40% in tumors in Mac-NG2ko mice (**Fig. 5F**). In addition, tumor vessels in Mac-NG2ko mice are 5-fold leakier than those in control tumors (**Fig. 5G**). Coupled with the reduced diameter of tumor vessels, these deficits in vascular function in Mac-NG2ko mice should result in a greatly diminished tumor blood supply, providing one possible explanation for the drastically reduced early tumor growth seen in these mice. Accordingly, we find that intratumoral hypoxia is increased 15-fold in Mac-NG2ko mice (**Fig. 5H**). As expected, increased hypoxia leads to elevated expression of HIF-1α (2-fold) in Mac-NG2ko tumors (**Fig. 5I**), with a corresponding 3-fold upregulation of VEGF-A expression (**Fig. 7G**). However, VEGF-A localization differs greatly in control and Mac-NG2ko tumors, being highly vascular in control tumors (**Figs. 7A–C**), vs. largely non-vascular in Mac-NG2ko tumors (**Figs. 7D–F**). Vascular VEGF-A is reduced 3-fold in Mac-NG2ko tumors (**Fig. 7H**), while non-vascular VEGF-A is elevated 5-fold (**Fig. 7I**).

## Discussion

Interactions between tumor cells and components of the host microenvironment are essential for supporting tumor growth and progression.^17-22^ Initially recruited as part of the host immune surveillance mechanism, immune cells make diverse contributions to multiple aspects of tumor progression. Macrophages, in particular, are efficiently subverted by tumor cells to performing a variety of tumor-promoting functions. Compared to other aspects of macrophage impact on tumor progression, such as immune suppression and promotion of tumor cell migration, invasion, and metastasis,^1,2^ the role of macrophages in promoting tumor vascularization is unexpectedly large.^3,4,23^ Macrophages can be extremely perivascular in nature,^8,24^ a property that may be important for both macrophage impact on tumor vessel development^7-9^ and vascular contributions to myeloid development.^25^

Our studies on pericyte-specific versus myeloid-specific ablation of the NG2 proteoglycan offer a case in point. Since pericytes are an integral component of microvessels, we expect interference with pericyte function to have significant negative effects on microvessel structure and function. Associated more peripherally with microvessels, macrophages with altered properties appear to be in a less advantageous position to affect microvessel function. Nevertheless, macrophage-specific ablation of NG2 results in more serious microvascular deficits than pericyte-specific NG2 ablation. **Table 1** shows that while PC-NG2ko mice and Mac-NG2ko mice exhibit a similar spectrum of structural and functional deficits in tumor vessels, vascular deficits in PC-NG2ko mice are invariably less severe than those seen in tumor vessels in Mac-NG2ko mice. Although in most cases these differences are a matter of degree, there are three instances in which the deficits are unique to tumor vessels in Mac-NG2ko mice. These are (a) the dramatic loss of N-cadherin expression by both pericytes and endothelial cells, (b) the marked loss of pericyte association with endothelial cells, and (c) the reduced diameter of tumor vessels. Changes in pericyte-endothelial cell interaction and in vessel diameter were previously noted in tumor vessels in germline NG2 null mice,^6,10^ but were not detected in tumor vessels in PC-NG2ko mice.^12^ N-cadherin expression was not examined in these previous studies.

**Table 1.** Differences in vessel structure and function between control and NG2 null mice

Parameter		Change (vs. control mice)		
		Germline-NG2ko mice	PC-NG2ko mice	Mac-NG2ko mice
Vessel structure				
	Pericyte coverage of endothelial cells	45% decrease	33% decrease	47% decrease
	Detached pericyte	200% increase	Unchanged	181% increase
	Vessel diameter	20% decrease*	Unchanged	53% decrease
	Basal lamina assembly	73% decrease	31% decrease	72% decrease
	N-cadherin (endothelial cell)	N.D.	Unchanged	74% decrease
	N-cadherin (pericyte)	N.D.	Unchanged	97% decrease
Vessel function				
	Vessel patency	50% decrease	43% decrease	48% decrease
	Vessel leakiness	400% increase	270% increase	416% increase
	Intratumoral hypoxia	2000% increase	560% increase	1580% increase

* Change in vessel diameter of spontaneous mammary tumors between control and global-NG2 ko mice.^6^

N.D. = not determined.

The sum of these qualitative and quantitative alterations in tumor vessels may at least partially explain why tumor growth is impacted more severely in Mac-NG2ko mice than in PC-NG2ko mice. It is important to emphasize that our current studies focus on early stages of tumor growth and vascularization. This is partly for practical reasons: structural and functional properties of vessels that we can successfully quantify at early time points become difficult to measure reproducibly at later time points, as tumor vessels become increasingly abnormal, and as tumors become increasingly necrotic. Nevertheless, the early time points represent a critical period during which successful tumor vascularization is a major factor in determining progression from hyperplasia to neoplasia.^26^ Thus, during this period, there may be a fairly direct correlation between effective tumor vascularization and tumor growth.

Significantly, pericyte-specific NG2 ablation and myeloid-specific NG2 ablation both seem to impact the same vascular target: namely, pericyte interaction with endothelial cells. In the case of pericytes, expression of NG2 is important for trans-activation of β1 integrin signaling in endothelial cells, promoting several aspects of endothelial cell function including the formation of endothelial cell junctions that enhance the barrier properties of the vascular endothelium.^12^ Although NG2-deficient pericytes still interact with endothelial cells, the association is less intimate, as judged by decreased physical overlap between the two cell types. This impairs the development of both cell types, leading to deficits in the assembly of the vascular basal lamina, increased vessel leakiness, and decreased vessel patency.^12^ In the case of macrophages, our results show that NG2 is important for macrophage recruitment to tumors. Mac-NG2ko results in very large deficits in the number of TAMs , a phenomenon not observed in PC-NG2ko mice. In the absence of macrophages, pericytes exhibit a greatly diminished ability to associate with endothelial cells, in spite of the fact that the pericytes still express NG2. The loss of a macrophage-derived signal, due to severely reduced macrophage recruitment, apparently disables a key mechanism required for pericyte-endothelial cell interaction.

One candidate for this mechanism is N-cadherin-mediated adhesion between pericytes and endothelial cells,^15,16^ an interaction that is important for several aspects of endothelial cell function, including expression of VE-cadherin that mediates endothelial junction formation.^14^ Macrophage deficits in tumors in Mac-NG2ko mice result in greatly decreased N-cadherin expression by both pericytes and endothelial cells, likely contributing to loss of interaction between these two vascular cell types. Decreased N-cadherin expression appears to be a cause of diminished pericyte-endothelial cell interaction rather than the result of diminished interaction, since loss of N-cadherin expression is not seen in PC-NG2ko mice, where reduced pericyte ensheathment of endothelial cells is also observed.^12^ The extent of pericyte ensheathment of endothelial cells in Mac-NG2ko mice is even lower than that observed in PC-NG2ko mice, likely underlying the more extreme changes in microvascular characteristics seen in the Mac-NG2ko mice. These include decreased pericyte maturation, reduced endothelial cell sprouting and junction formation, and diminished assembly of the vascular basement membrane. Additional work will be required to understand the impact of lost N-cadherin-mediated interactions between pericytes and endothelial cells. The crosstalk known to exist between N-cadherin and β1 integrin signaling^27,28^ makes this an especially intriguing topic in light of the role of NG2 in promoting β1 integrin activation in endothelial cells. Information is also needed regarding the macrophage-dependent signals that control N-cadherin expression in the two microvascular cell types. In this context, it is interesting that a soluble (non-VEGF-A) factor(s) derived from microglia is required for vessel sprouting in a cell culture model of angiogenesis.^29^ An interesting candidate in this regard is TGF-β, a product of TAMs^30,31^ that has the ability to activate Smad4 in endothelial cells, resulting in upregulation of N-cadherin and stabilized endothelial cell interaction with pericytes.^32^

The observed structural changes in tumor blood vessels in Mac-NG2ko mice lead to diminished functional properties of these vessels. For example, vessel patency is reduced, while vessel leakiness is increased, resulting in reduced blood flow to Mac-NG2ko tumors, increased intratumoral hypoxia, and upregulation of HIF-1α and VEGF-A expression. Although we might expect increased levels of VEGF-A to promote vascular development, in fact, the diameter of tumor vessels in Mac-NG2ko mice is reduced 2-fold compared to controls. This may be due in part to the fact that VEGF-A in Mac-NG2ko tumors is largely not associated with vessels, while VEGF-A localization in control tumors is highly vascular. The loss of vascular VEGF-A localization may be attributable to diminished assembly of the vascular basal lamina in vessels in Mac-NG2ko tumors. The importance of ECM-mediated VEGF sequestration for vessel development has been previously noted.^33^ We have reported a similar loss of vascular localization of VEGF in B16F10 tumors grown in collagen VI null mice^11^ and in MMTV-PyMT mammary tumors in NG2 null mice.^6^ As in the case of Mac-NG2ko mice, the loss of vascular VEGF localization in these previous studies was correlated with greatly reduced basal lamina assembly. In each case, reduced vessel diameter is the most dramatic structural manifestation of decreased vascular VEGF localization.

Of equal interest are mechanisms by which NG2 promotes macrophage recruitment to tumors. Similar to the loss of NG2-dependent macrophage infiltration of tumors, we have also reported that NG2 ablation greatly reduces macrophage recruitment to demyelinated lesions in mouse spinal cord,^34^ suggesting the generality of NG2 involvement in macrophage mobilization to tissues. In both the demyelination and tumor studies, transplantation of EGFP-positive bone marrow establishes that the myeloid cells in question are primarily macrophages rather than tissue-resident microglia. Since myeloid-specific NG2 ablation does not decrease the overall number of circulating macrophages, an attractive alternative mechanism is that NG2 is important for macrophage extravasation from vessels. While the cascade of leukocyte rolling, arrest, crawling, and membrane transmigration is extremely complex, the established importance of macrophage α4β1 integrin interaction with endothelial cell VCAM-1 in this process^35,36^ suggests a possible mechanism for NG2 involvement. This is based on the ability of NG2 to enhance β1 integrin signaling in several cell types, including endothelial cells, pericytes, and tumor cells.^12,37-39^ Ablation of NG2 might diminish α4β1 activation in macrophages, weakening α4β1 interaction with endothelial VCAM-1 to the extent that extravasation is impaired. Another possibility is suggested by the fact that recruitment of CD11b (αM integrin)-positive and CD18 (β2 integrin)-positive macrophages is strongly diminished by NG2 ablation. The αMβ2 integrin heterodimer is another important player in leukocyte extravasation via its interaction with endothelial cell ICAM-1.^25,36,40^ The αMβ2-ICAM-1 interaction might also be weakened by macrophage loss of NG2, assuming that NG2 activates β2 integrins via a mechanism similar to that seen with β1 integrins. It remains for us to establish the ability of NG2 to promote the required activation of β2 integrins.

Our studies of late-stage tumors (day 16) demonstrate that while NG2 expression enhances macrophage extravasation into tumors, the proteoglycan is not absolutely required for this process. Although slowed by NG2 ablation, macrophage recruitment to tumors in Mac-NG2ko mice eventually approaches levels seen in control mice. Increased macrophage numbers are accompanied by normalization of pericyte interactions with endothelial cells, such that tumor vessel diameter in Mac-NG2ko mice is similar to that seen in control mice. This results in the surprising observation that survival is not significantly improved by Mac-NG2ko, while a small survival advantage is achieved by pericyte-specific NG2 ablation.

Since we have only examined vascular function, we must consider that other NG2-dependent macrophage functions may also be important in promoting tumor progression. For example, in the polyoma middle T transgenic mouse model of breast cancer, we have demonstrated that germline ablation of NG2 affects not only tumor vascularization, but also aspects of macrophage phenotype that may be relevant to M1–M2 polarization.^6^ We have also reported that NG2 ablation affects the expression of pro-inflammatory and anti-inflammatory cytokines in a tissue-specific manner.^13,34^ Classically-activated M1 macrophages produce pro-inflammatory cytokines and can have antitumor functions. Conversely, TAMs frequently exhibit an alternatively-activated M2 phenotype, producing anti-inflammatory cytokines that promote tumor growth.^1^ Effects of NG2 on macrophage polarization are therefore likely to be highly relevant to macrophage impact on tumor progression. These possibilities remain to be investigated.

The importance of vascularization for tumor growth along with the expression/activity of NG2 in both pericytes and macrophages suggests that the proteoglycan can be a key stromal factor in the development of all solid tumors. NG2 expression by pericytes is low in quiescent vasculature, but is quickly upregulated in any situation that involves vascular remodeling. Thus, we expect NG2 levels to be high on pericytes in tumor vessels due to the dynamic nature of tumor vasculature. Although NG2 expression by macrophages is poorly understood, our experience suggests that the proteoglycan is not expressed by quiescent monocytes/macrophages, but is quickly upregulated upon activation of myeloid cells by signals such as ligand binding to toll-like receptors. The fact that macrophage expression of NG2 is quite transient makes it difficult to perform certain types of quantitative studies on NG2-positive populations. NG2 expression, even though transient, is nevertheless sufficient to transform the properties of these cells during early stages of recruitment and extravasation. The universality of macrophage infiltration in all types of inflammatory situations indicates that NG2 may represent an important aspect of macrophage recruitment in multiple pathologies, including cancer. These findings emphasize the possible value of targeting pericytes and macrophages as a means of improving cancer therapy.

In our studies, we have utilized NG2-negative B16F10 melanoma cells in order to focus on stromal roles of NG2. However, the proteoglycan is also expressed by tumor cells in several types of cancer, including melanoma,^41-44^ glioblastoma,^38,45,46^ sarcoma,^47^ myeloid leukemia,^48^ and triple-negative breast cancer.^49^ In all cases, increased NG2 expression by tumor cells is associated with enhanced tumor progression and worsened patient prognosis. Tumor cell NG2 promotes neoplastic progression by enhancing tumor cell proliferation, motility, and survival via enhanced integrin and growth factor signaling.^50^ This amplifies the potential benefit of targeting these tumors for NG2 as a multi-focal component of both the tumor and the tumor stroma.

## Materials and Methods

### Cell lines

NG2-negative B16F10 melanoma cells (C57Bl/6)^10-12^ were maintained in Dulbecco's Modified Eagle's Medium (DMEM; GIBCO by Life Technologies, 12800-017) containing 10% fetal bovine serum. The absence of NG2 on the B16F10 cells allows us to focus on stromal roles of the proteoglycan in tumor progression. Moreover, since we have used B16F10 cells in three previous studies of brain tumor progression,^10,11^ continued use of these cells permits useful comparison of current and previous data. This is especially important for comparing the vascular phenotypes of PC-NG2ko mice ^12^ and Mac-NG2ko mice (current study).

### Animals and tumor implantation

Mice were maintained in the Sanford-Burnham Vivarium (fully accredited by the Association for Assessment and Accreditation of Laboratory Animal Care). All animal procedures were performed in accordance with Office of Laboratory Animal Welfare regulations and were approved by Sanford-Burnham Institutional Animal Care and Use Committee review prior to execution. NG2 floxed mice,^13^ pdgfrb-Cre transgenic mice,^12,51^ and lysozyme M-Cre (LysM-Cre) transgenic mice^52,53^ were maintained on C57Bl/6 backgrounds. Syngeneic intracranial B16F10 tumors were established as previously described.^10-12^ In general, cohorts of from five to ten mice were used in each of the studies described below. At 10 d after injection of B16F10 cells, animals under Avertin anesthesia were perfused with 1% paraformaldehyde prior to dissection of brains. Exceptions to this regimen were cases in which mice were injected with FITC-dextran, FITC-Lycopersicon esculentum agglutinin (LEA) lectin, or pimonidazole. After cryoprotection in 20% sucrose, fixed brains were frozen in OCT compound and cryosectioned (20 μm) for analysis by immunostaining. In some cases, the survival times for tumor-bearing PC-NG2ko and Mac-NG2ko mice were compared with survival times for control mice. On the day prior to death (judged by mouse appearance and behavior), animals were euthanized by perfusion with 1% paraformaldehyde under Avertin anesthesia. Specimens for study were taken from mice that survived until day 16 after tumor initiation.

### Bone marrow transplantation and flow cytometry

Bone marrow transplantations from male β-actin-EGFP transgenic donors (C57Bl/6; Jackson Labs) were carried out as previously described.^6^ Wild type recipients at 8 weeks of age were gamma irradiated with two doses of 5 Gy each, administered 3 h apart, and were immediately reconstituted by retro-orbital injection of 7 × 10^5^ bone marrow cells in 100 μL of PBS containing 2% mouse serum. Parallel transplantations were performed with EGFP+ NG2^+/+^ and EGFP+ NG2^−/−^ bone marrow. Engrafted mice were maintained on antibiotic water (2.2 g/L neomycin sulfate and 13 mg/L Polymyxin B) for 6 weeks. Peripheral blood samples were collected 6 weeks after transplantation for FACSCanto flow cytometric analysis of the extent of EGFP engraftment. Animals with at least 75% engraftment were utilized for the analysis of macrophage populations shown in **Figure 3**.

Heparinized whole blood was collected from control and Mac-NG2ko mice for quantification of circulating macrophages. Following treatment with ACK buffer to lyse red blood cells, incubations with APC-labeled CD11b antibody (eBioscience, San Diego, CA) and PE-Cy5-labeled F4/80 antibody (Biolegend, San Diego, CA) were carried out for 30 minutes in the cold. Washed cells were processed for flow cytometric analysis using the FACSCanto instrument.

### Immunohistochemistry and confocal microscopy

Macrophage recruitment and perivascular localization were evaluated using rabbit anti-NG2 (1:100),^10,11^ hamster anti-mouse CD31 (1:100; Thermo Scientific, by Life Technologies, MA3105), rat anti-mouse CD11b (1:100, BD PharMingen, 550282), rat anti-mouse F4/80 (1:100, Molecular Probes by Life Technologies, MF48000), and rat anti-mouse CD18 (1:100, eBioscience, 14-0181). NG2 ablation in macrophages vs. pericytes was quantified using guinea pig anti-NG2 (1:100),^12^ rabbit anti-PDGFRβ (1:100),^10,11^ rat anti-mouse CD11b (1:100), and rat anti-mouse F4/80 (1:100). Pericyte ensheathment of endothelial cells was assessed using rabbit anti-PDGFRβ (1:100) and rat anti-mouse CD31 (1:100; BD Pharmingen, 553370). Pericyte maturation was quantified using mouse anti-α-smooth muscle actin (α-SMA, 1:500; Sigma–Aldrich, F3777) and rabbit anti-PDGFRβ (1:100). Vascular basement membrane assembly was assessed using rabbit anti-collagen IV (1:100, Millipore, AB756P) and rat anti-mouse CD31 (1:100). Endothelial cell sprouting was examined using goat anti-mouse VEGFR3 (1:500, R&D Systems, AF743), rat anti-mouse CD31 (1:100), and rabbit anti-mouse LYVE-1 (1:500, AngioBio Co., 11-034). N-cadherin expression by endothelial cells or pericytes was assessed using rabbit anti-N-Cadherin (1:250, abcam, ab18203) with rat anti-mouse CD31 (1:100) or goat anti-mouse PDGFRβ (1:25, LifeSpan BioSciences, LS-C150228), respectively. Endothelial cell junction formation was quantified using rat anti-mouse CD144 (VE-Cadherin, 1:50, BD PharMingen, 550548) and hamster anti-mouse CD31 (1:100). Vascular patency and leakage were determined as previously described,^10-12^ using intravenously injected FITC-labeled LEA lectin (Vector Laboratories, FL-1171, 0.1 mL of 0.5 mg/mL solution) or FITC-labeled dextran (250 kDa; Sigma–Aldrich, FD-250S, 0.1 mL of a 50-mg/mL solution). Tumor-bearing brains were removed without fixative perfusion and fixed overnight in 1% paraformaldehyde. Labeling of vessels with FITC-labeled LEA lectin or FITC-labeled dextran was compared with immunolabeling for rat anti-mouse CD31 (1:100). Tumor hypoxia was evaluated with a Hypoxyprobe-1 kit (Hypoxyprobe, Inc.). Tumor-bearing mice were injected intravenously with 60 mg/kg pimonidazole hydrochloride and then perfused with 1% paraformaldehyde after a 60-min incubation period. For visualization of hypoxic areas, sections were incubated overnight with FITC-conjugated mouse anti-pimonidazole hydrochloride (1:100; Hypoxyprobe, Inc.) antibody and rabbit anti-HIF-1α (1:100).^11^ VEGF-A expression in tumors was detected by goat anti-mouse VEGF-A (1:50, R&D Systems, AF-493-NA) and rat anti-mouse CD31 (1:100). Secondary antibodies included FITC-, Cy3-, or Cy5-labeled (1:250, Jackson ImmunoResearch Laboratories Inc.), and Alexa 488-, Alexa 568 or Alexa 647-labeled (1:250; Invitrogen by Life Technologies) goat, donkey, or mouse anti-rat, anti-hamster, anti-rabbit, anti-goat, or anti-mouse IgG.

Examination and image capture (TIFF images) from immunostained tumor sections were accomplished using a Fluoview 1000 Laser Point Scanning Confocal Microscope (Olympus, Tokyo, Japan) as described previously.^10-12^ Serial 1 μm optical sections were scanned across the entire 20μm thickness of the histologic specimens. Areas (number of pixels) with immunostaining greater than a set threshold were quantified using computer based morphometry software (Image-Pro Plus 4.5, Media Cybernetics Inc., Bethesda, MD). Data are derived from four independent tumors of each genotype, sampling four fields from three sections in each tumor. In several cases we used z-stacks of confocal images to quantify the spatial relationship/overlap between markers that are not present on the same populations of cells or structures (for example, pericytes and endothelial cells, macrophages and endothelial cells, endothelial cells and vascular basement membrane, and endothelial cells and VEGF). Although these marker pairs cannot overlap with each other in single optical sections, they nevertheless appear to overlap when viewed in 3-dimensional space. Analysis of z-stacks of confocal images therefore allowed us to quantify the extent of marker overlap in 3-dimensions, as a measure of physical interaction.

### Statistical analysis

All results are expressed as mean ± SE. Statistical analyses were performed using the two-tailed *t* test. *p* < 0.05 was considered statistically significant.
