# Supplementary material for: NG2 proteoglycan-dependent recruitment of tumor macrophages promotes pericyte-endothelial cell interactions required for brain tumor vascularization
Source: Oncoimmunology. 2015 Jan 22;4(4):e1001204. doi: 10.1080/2162402X.2014.1001204 (PMC4485789; doi:10.1080/2162402X.2014.1001204)
Supplement: Supplementary_files.zip [file koni-04-04-1001204-s001.zip › 1001204_Supplementary Materials/1001204 Supplemental Figures.pdf]

< Supplementary Figures >

Title: NG2 proteoglycan-dependent recruitment of tumor macrophages promotes pericyte-endothelial cell interactions required for brain tumor vascularization.

Authors and affiliations:

Fusanori Yotsumoto[1,4], Weon-Kyoo You[1,2], Karolina Kucharova[1], Kenji Sakimura[3], and William B. Stallcup [1]

[1] Sanford-Burnham Medical Research Institute, Cancer Center, 10901 North Torrey Pines Road, La Jolla, CA 92037, USA.

[2] current address: Biologics Business, Research and Development Center, Hanwha Chemical, 76 Gajeong-Ro Yuseong-Gu, Daejeon 305-804, South Korea.

[3] Department of Cellular Neurobiology, Brain Research Institute, Niigata University, Niigata 951-8585, Japan.

[4] current address: Department of Biochemistry, Faculty of Medicine, Fukuoka University, Fukuoka 814-0180, Japan.

Corresponding author: Dr. Fusanori Yotsumoto, MD, PhD.

Sanford-Burnham Medical Research Institute, Cancer Center, Tumor Microenvironment and Metastasis Program, 10901 North Torrey Pines Road, La Jolla, CA 92037, USA; Tel: (858) 646-3100 x3220; Fax: (858) 646-3197; e-mail: fyotsumoto@sanfordburnham.org

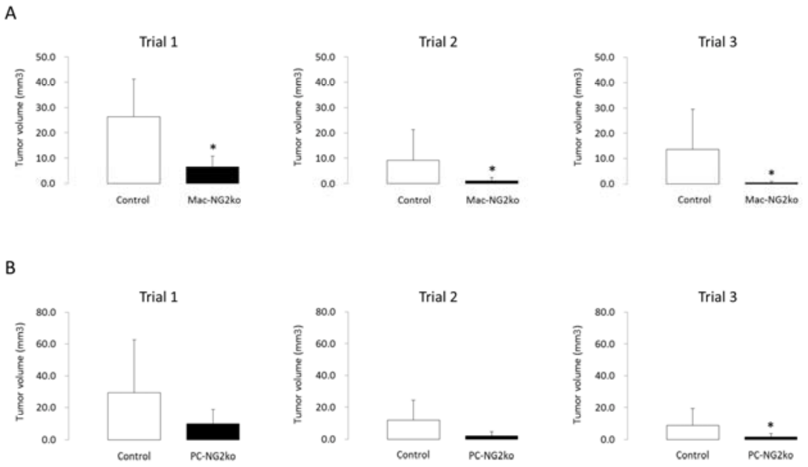

Supplementary Figure 1. Decreased brain tumor progression in myeloid- and pericyte-specific NG2 null mice: individual trials

(A) Quantification of 10-day tumor volumes in control and Mac-NG2ko mice. Trial 1 (control: N=8, Mac-NG2ko: N=5). Trial 2 (control: N=5, Mac-NG2ko: N=5). Trial 3 (control: N=8, Mac-NG2ko: N=10). Macrophage-specific NG2 ablation significantly reduced tumor volumes in all three trials. (B) Quantification of 10-day tumor volumes in control and PC-NG2ko mice. Trial 1 (control: N=10, PC-NG2ko: N=7). Trial 2 (control: N=7, PC-NG2ko: N=6). Trial 3 (control: N=5, PC-NG2ko: N=5). Pericyte-specific NG2 ablation also reduced tumor growth, although statistical significance was achieved only in Trial 3. *P<0.01 compared to controls.

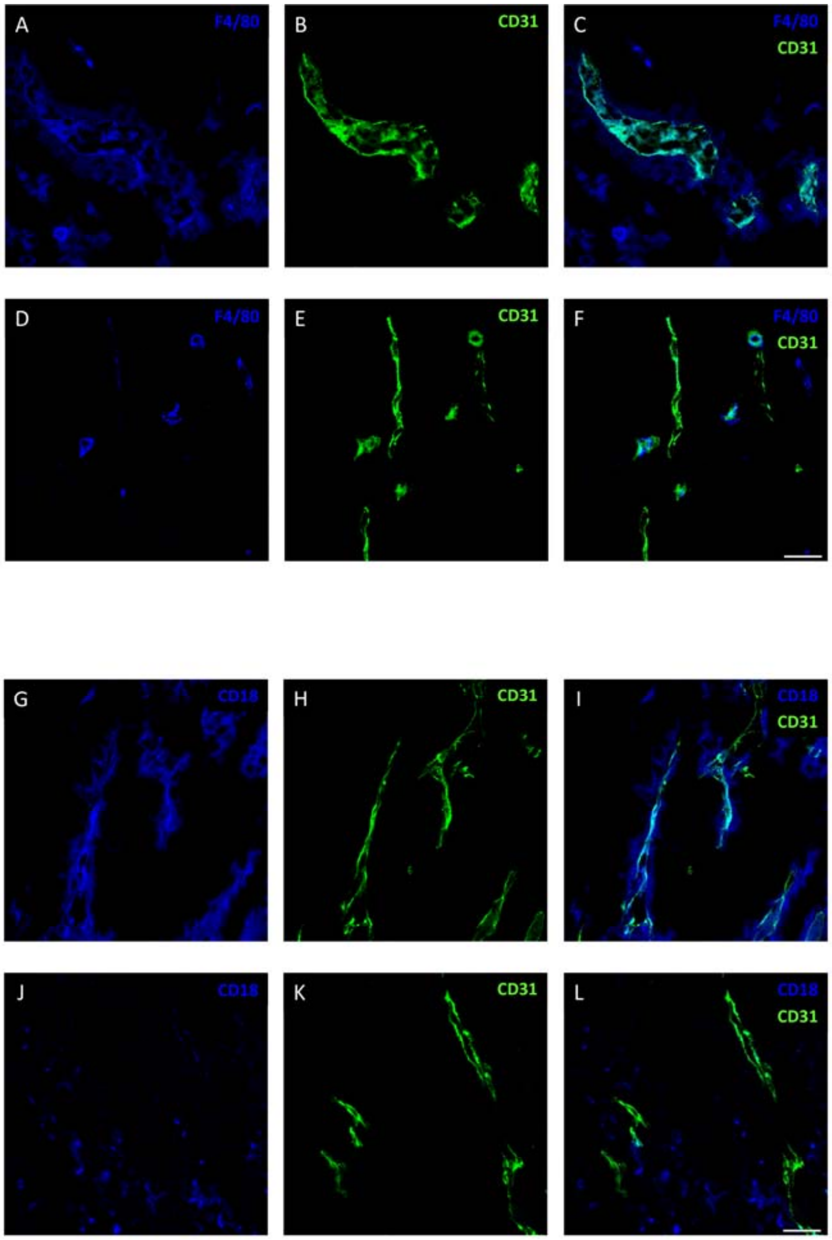

Supplementary Figure 2. Loss of F4/80 or CD18 macrophage recruitment following myeloid-specific NG2 ablation

Double immunostaining for F4/80 (A-F) or CD18 (G-L) (blue) and CD31 (green) in tumor sections from control (A-C; G-I) and Mac-NG2ko mice (D-F; J-L). Areas of F4/80-CD31 or CD18-CD31 overlap appear as pale blue. Scale bars = 20 μm.

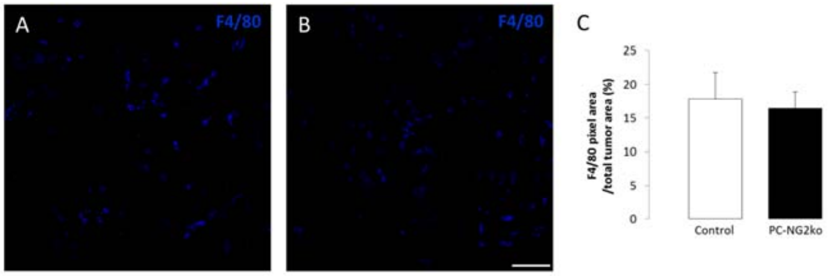

Supplementary Figure 3. Unaltered macrophage recruitment in PC-NG2ko mice

Immunostaining for F4/80 (blue) in tumor sections from control (A) and PC-NG2ko mice (B). (C) Quantification of macrophage abundance in tumors using the marker F4/80. Data are plotted as the percentage of total tumor area occupied by marker pixels. Scale bar = 60 μm.

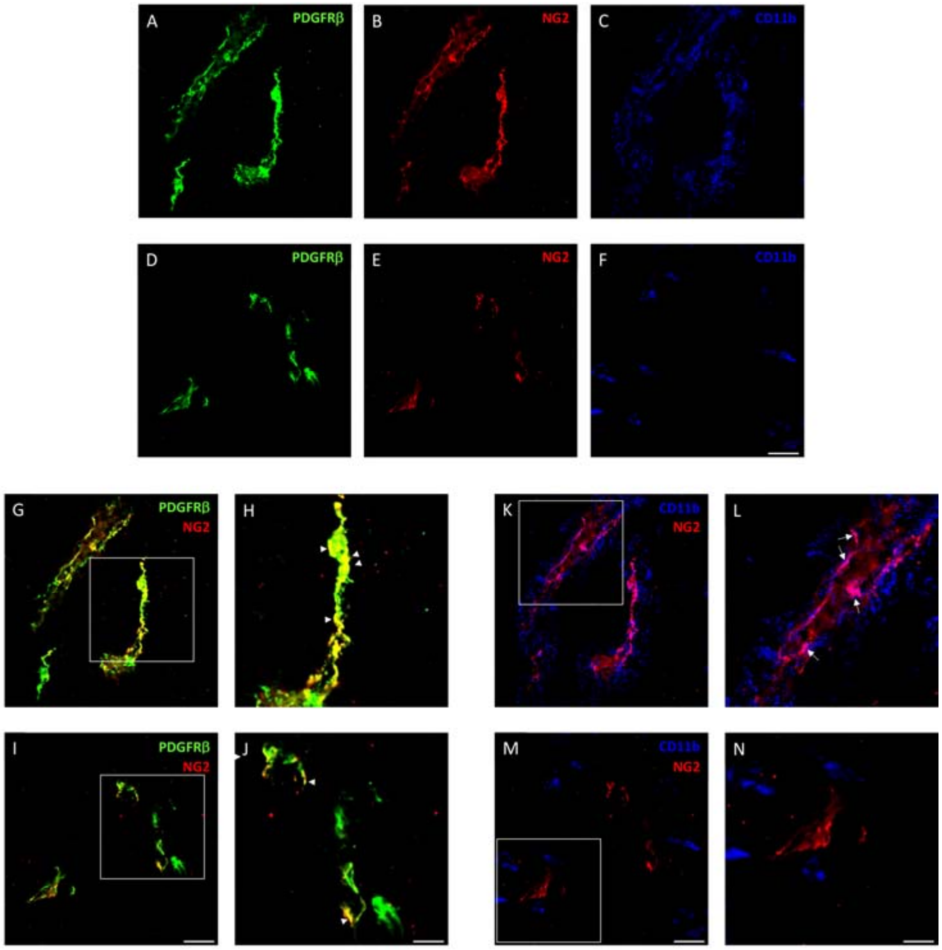

Supplementary Figure 4. Specificity of NG2 ablation in macrophages

Triple immunostaining for PDGFRβ (green), NG2 (red), and CD11b (blue) was used to assess NG2 expression (B,E) by pericytes (A,D; G-J) and macrophages (C,F; K-N) in tumor sections from control (A-C; G,H; K,L) and Mac-NG2ko mice (D-F; I,J; M,N). Panels A-F show images of single labeling. Panels H, J, L, and N are high magnification images of double-labeling in single optical sections from the boxed areas in G, I, K, and M, respectively. NG2 is expressed by PDGFRβ-positive pericytes in both control and Mac-NG2ko tumors (arrowheads in H and J). NG2 is present on macrophages in control tumors (arrows in L), but is absent from macrophages in Mac-NG2ko tumors (N). Scale bars = 20 μm in F, I and M and 10 μm in J and N.

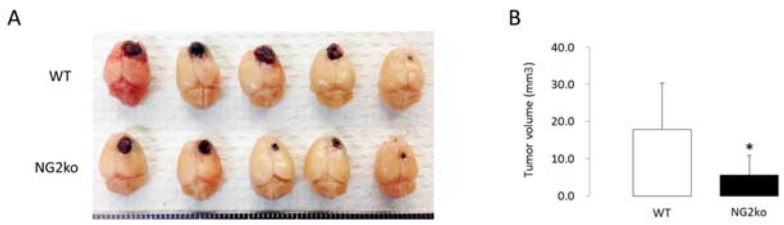

Supplementary Figure 5. Brain tumor growth in bone marrow transplanted mice

Representative images (A) and bar graphs (B) illustrate the differences in volume of B16F10 melanoma tumors after 10 days of growth in the brains of mice transplanted with bone marrow cells from wild-type or NG2 null EGFP mice. n = 5 for both sets of mice. *P<0.05 versus control.

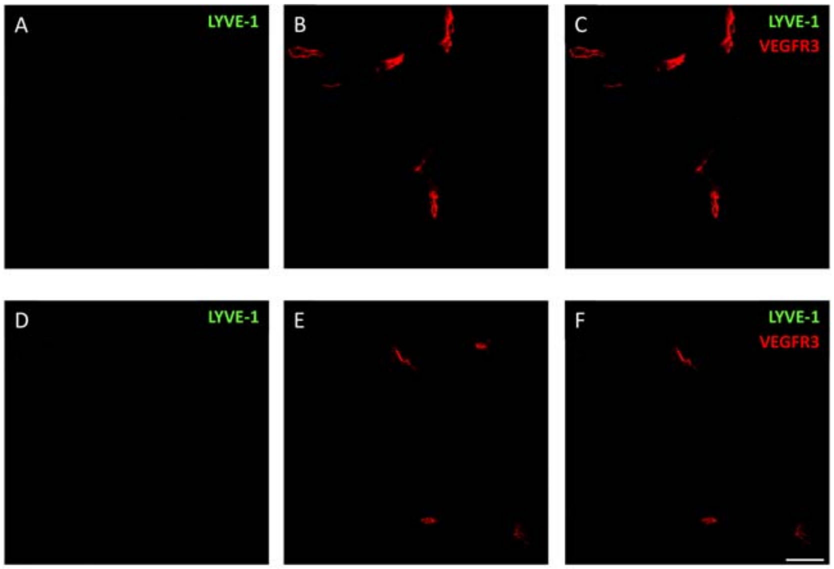

Supplementary Figure 6. Specificity of VEGFR3 expression by sprouting endothelial cells

Double immunostaining for LYVE-1 (green) and VEGFR3 (red) was used to demonstrate the absence of lymphatic endothelial cells in control (A-C) and Mac-NG2ko tumors (D-F). Panels C and F are merged images. The absence of LYVE-1 labeling demonstrates that VEGFR3 labeled cells are sprouting endothelial cells rather than lymphatic endothelial cells. Scale bar = 20 μm.

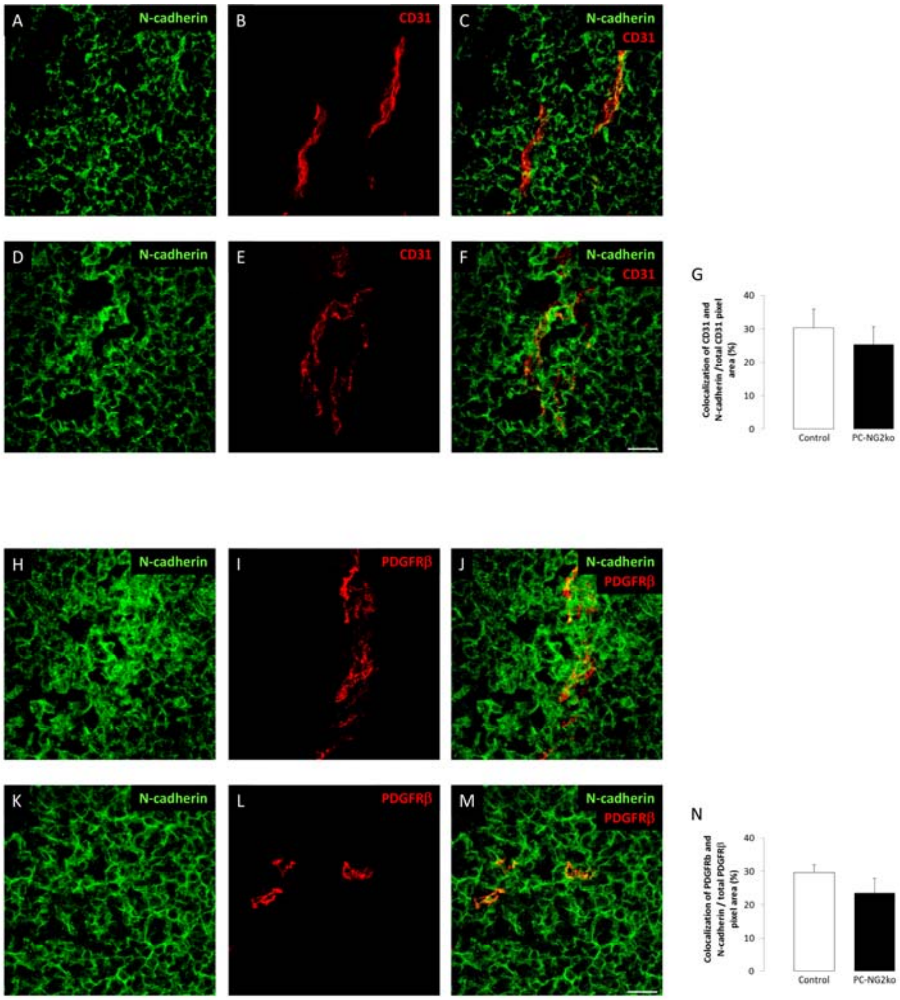

Supplementary Figure 7. Unchanged N-cadherin expression following pericyte-specific ablation of NG2

Double immunostaining for CD31 (red) and N-cadherin (green) was used to assess endothelial cell expression of N-cadherin in control (A-C) and PC-NG2ko tumor vessels (D-F). (G) Quantification of N-cadherin/CD31 colocalization as a percentage of total CD31. Double immunostaining for PDGFGRβ (red) and N-cadherin (green) was used to evaluate pericyte expression of N-cadherin in control (H-J) and PC-NG2ko tumor vessels (K-M). (N) Quantification of N-cadherin/PDGFRβ colocalization as a percentage of total PDGFRβ. Scale bars = 20 μm.
